# The effect of omega-3 on serum lipid profile in hemodialysis patients

**DOI:** 10.12861/jrip.2015.14

**Published:** 2015-09-01

**Authors:** Hamid Reza Omrani, Yahya Pasdar, Daryosh Raisi, Farid Najafi, Ardeshir Esfandiari

**Affiliations:** ^1^Department of Internal Medicine, Imam Reza Hospital, Kermanshah University of Medical Sciences, Kermanshah, Iran; ^2^Department of Nutrition, School of Public Health, Kermanshah University of Medical Sciences, Kermanshah, Iran; ^3^Department of Internal Medicine, Kermanshah University of Medical Sciences, Kermanshah, Iran; ^4^Research Center of Environmental Determinant of Health, Kermanshah University of Medical Sciences, Kermanshah, Iran; ^5^Department of Internal Medicine, Kermanshah University of Medical Sciences, Kermanshah, Iran

**Keywords:** Omega 3, Hemodialysis, Serum lipids

## Abstract

**Introduction: ** One of the major complications in hemodialysis patients is cardiovascular disease. Lipid abnormality is an important factor in the development of cardiovascular disease.

**Objectives:** To investigate the effect of omega-3 supplementation on serum lipid profile in hemodialysis patients.

**Patients and Methods:** This randomized clinical trial study included 2 groups of patients: those who received omega-3 supplementation (experimental group; 30 cases) and those who received placebo (control group; 30 cases). They received these for 10 weeks and serum lipid profile (triglyceride, high-density lipoprotein [HDL-C], low-density lipoprotein [LDL-C], and total cholesterol) was assessed 2 times, first before initiating supplementation and then at the end of 10-week study period.

**Results: ** Mean (±SD) serum total cholesterol levels at the beginning was 163 (±47) mg/dL in experimental group and 143 (±25) mg/dL in control group (P> 0.05). These values decreased to 124 mg/dL and 132 mg/dL in control and experimental groups (P< 0.05). There was no significant difference of HDL-C, LDL-C, or triglyceride levels between experimental and control groups after 10 weeks of treatment.

**Conclusion:** Omega-3 supplementation in hemodialysis patients only showed significant decrease of serum total cholesterol level, but not other lipids.

**Implication for health policy/practice/research/medical education:**

Since there is controversy about omega-3 supplementation on lipid profile in patients with hemodialysis patients, we conducted a study to determine the role of omega-3 in such patients. Our results showed that in short-term follow-up omega-3supplementation, the only finding was significant decrease in serum total cholesterol.

## Introduction

The most significant cause of death among hemodialysis patients is cardiovascular disease and one of its primary causes is coronary artery atherosclerosis (1). High concentration of serum lipid, as previously demonstrated in numerous studies, has been considered an independent risk factor for atherosclerosis development (2). With regard to impaired serum lipid profile in hemodialysis patients, this in turn exacerbates development of cardiovascular diseases in these patients. Hence, control, monitoring, and treatment of serum lipid abnormalities can be a necessity for prevention of related mortality in these patients (3-5). Nevertheless, given that lipid-lowering medications alone may not be effective to correct abnormalities of lipid and also considering the side effects of these medications such as gastrointestinal distress, flushing, itching, myositis, and hepatic dysfunction as well as digestive disorders and uremic pruritus in these patients (6,7), it seems logical to find other methods of preventing and treating hyperlipidemia besides existing medications.

In this regard, several studies have been taken to find agents to manage lipid abnormalities (8-10). One of these is the effect of supplementation with omega-3 or consuming foods containing omega-3. Fatty acid omega-3 has numerous benefits for hemodialysis patients (11-14). However, some studies did not demonstrate beneficial effects of using omega-3 supplementation in terms of lipid profile in hemodialysis patients (15,16).

With regard to the special geographical position of Kermanshah province which is located in a mountainous area and lack of easy access to the marine resources which are rich in omega-3, as well as not regular consumption of fish in this province, supplementation with omega-3, if demonstrated to be effective in reducing serum lipid levels, will have beneficial effects for hemodialysis patients.

## Objectives

The aim of this study was to assess the effects of omega-3 on the serum lipid profile in hemodialysis patients.

## Patients and Methods

This study was double-blind clinical trial performed on patients undergoing hemodialysis and presented to the hemodialysis center Imam-Reza hospital in Kermanshah in 2013 (from October for 10 weeks).

Inclusion criteria were satisfaction and cooperation and their willingness to be studied and passing at least 6 months from the onset of dialysis. Exclusion criteria included the presence of bleeding and taking warfarin, ticlopidine, dipyridamole and clopidogrel, viral hepatitis and other infectious diseases, body mass index (BMI) of less than 18.5 or more than 30 kg/m^2^ and supplementation with omega-3, omega-6 and vitamins E and C within the two months preceding the study.

To start the study, 5 cc venous blood in the fasting state and before connection to hemodialysis machine was obtained. After completion of hemodialysis, weight with light clothing using a weight scale with precision of 100 g was measured. Height was also measured without shoes by a meter installed on the wall with precision of 1 mm.

General characteristics of the patients were recorded on data collection sheets. Blood pressure was recorded 2 times (before and after hemodialysis) using sphygmomanometer.

In this study, patients were randomly divided into 2 groups of receiving omega-3 supplementation or placebo.

Patients in omega-3 group (experimental group) took omega-3 for 10 weeks (1000 mg daily) as capsules containing 80 mg of EPA and 120 mg DHA. Patients in placebo group received vitamin E in the form of capsule which was similar in shape and size to omega-3 capsule (one capsule daily).

For more serious implementation of programs and conducting research compliance in the course of a double-blind placebo, at the beginning of the study a set of cans containing supplement or a placebo were coded by a person other than those involved in the study (measuring height and weight and providing the drugs) in the form of A and B. This was done as the researcher could not understand of the study groups. At the end of week 10 of the study, 5 cc blood was obtained before connecting to dialysis machine. Weight and height were also recorded after hemodialysis session. Furthermore, for better and more accurate taking of medications, every night at 9 pm the cases were announced by sending SMS to remind them taking their medications.

Biochemical parameters measured included total cholesterol, triglyceride, low-density lipoprotein (LDL-C), and high-density lipoprotein (HDL-C). These were measured once before starting the study and again after 10 weeks and before connection to hemodialysis machine. The fasting time recommended for patients was 12 hours.

Study population consisted of hemodialysis patients presented to the dialysis center. They were divided into 2 groups of receiving omega-3 or placebo. All the information needed was extracted from a form designed for this study. Sampling method was convenience method. The purpose of this study was to compare experimental and control groups. The sample size was calculated using triglyceride and 25 subjects were selected for each group. Thirty patients were selected so that if there would be drop or some patients will not complete the study, we can achieve 25 subjects. Totally 60 patients were entered into the study. Considering 99% confidence and 95% power, mean (standard deviation) of experimental and control groups was 246 (±25) and 276.7 (±41), respectively and the minimum sample size was 25 cases in each group.

### Ethics issues

The research followed the tenets of the Declaration of Helsinki. Informed consent was obtained from all patients. Omega-3 and placebo were free and the results were provided for them. In cases where lipid abnormality was severe and the patients required multi-dimensional treatments (diet, supplements, and medication), necessary treatments were delivered after completion of the study under supervision of dietician and nephrologist. The research was approved by the Ethics Committee of Kermanshah University of Medical Sciences.

### Statistical analysis

After collecting the data, they were entered into the SPSS software (version 20.0) and for summarizing the results, descriptive indices such as mean, standard deviation, and one-dimensional and 2-dimensional tables were utilized. To analyze the continuous data, independence and paired *t* test and Stata software were applied and the maximum acceptable error less than 0.05 was set as significance level.

## Results

In this study, in a time period of 10 weeks, 60 patients were divided into 2 groups of experimental group and control group, each 30 cases. In experimental group, there were 13 females and 16 males. In control group, there were 10 females and 19 males (*P* > 0.05). There was one death in experimental group and one patient immigrated in control group which led to remaining of 29 patients in each group.

Mean age of patients in experimental group was 55 years and in control group it was 56 years (*P* > 0.05).

Mean weight of experimental and control group patients before starting the study was 68 kg and 63 kg, respectively which changed to 69 kg and 64 kg (*P* > 0.05; Table 1).

**Table 1:** Comparison of the studied variables between experimental and control groups at 2 different time points (baseline and at the end of the study

**Variable**	**Baseline**			**End of the Study**		
	**Experimental Group**	**Control Group**	***P***	**Experimental Group**	**Control Group**	***P***
Age, y	55	56	> 0.05	-	-	-
Weight, kg	68	63	0.06	69	64	0.07
BMI, kg/m^2^	23.5	22.5	0.1	-	-	-
BP, mm Hg	128	131	0.6	126	132	0.1
Chol, mg/dL	163	143	0.55	124	132	0.45
TG, mg/dL	182	161	0.24	165	126	0.94
HDL-C, mg/dL	30	30	0.44	32	38	0.93
LDL-C, mg/dL	104	105	0.54	106	100	0.14

Mean height of patients before and after study was 170 cm in experimental group and 167 cm in control group (*P* > 0.05).

Mean blood pressure in experimental group was 128 mm Hg and in control group was 131 mm Hg at the start of the study (*P* = 0.06; Table 1). At the end of the study, mean blood pressure changed to 126 mm Hg in experimental group and 132 mm Hg in control group (*P* > 0.05).

Mean BMI of patients was 23.5 kg/m2 in experimental group and 22.5 kg/m2 in control group (*P* = 0.1; Table 1).

Mean (±SD) serum total cholesterol levels at the beginning were 163 (±47) mg/dL in experimental group and 143 (±25) mg/dL in control group (*P* > 0.05). These values decreased to 124 mg/dL in experimental group and 132 mg/dL in control group (*P* < 0.05; Table 1).

Mean (±SD) serum triglyceride levels at the beginning were 182 (±17) mg/dL in experimental group and 161 (±21) mg/dL in control group (*P* = 0.54). These values decreased to 165 mg/dL in experimental group and 126 mg/dL in control group (*P* > 0.05; Table 1).

Mean (±SD) serum HDL-cholesterol levels at the beginning were 30 (±9) mg/dL in experimental group and 30 (±8) mg/dL in control group (*P* = 0.8). These values changed to 32 mg/dL in experimental group and 38 mg/dL in control group (*P* > 0.05; Table 1).

Mean (±SD) serum LDL-cholesterol levels at the beginning were 104 (±25) mg/dL in experimental group and 105 (±21) mg/dL in control group (*P* = 0.9). These values changed to 106 mg/dL in experimental group and 100 mg/dL in control group (*P* > 0.05; Table 1).

In addition to the above mentioned serum markers, other tests including serum albumin, liver function tests (AST, ALT, alkaline phosphatase) and prothrombin time were also studied which did not show significant changes at the end of the study compared to the beginning.

## Discussion

Hemodialysis patients due to end-stage renal disease (ESRD) develop this condition due to various causes. Diabetes is one of the most prevalent contributing factors. These patients lose their renal function and to survive need hemodialysis or renal transplantation. On the other hand, most patients experience worsening of their condition due to complications of renal failure or a chronic disease damaging kidney function or other comorbidities. This process also induces acute or chronic mental problems including depression. This in turn makes management of these patients difficult. One of the main causes of death in these patients is cardiovascular problems and, accordingly, any factor that predisposes to cardiovascular events is a life threatening factor in this population (2,3).

The current study addresses one of the contributing factors in development of coronary heart disease, which is lipid abnormality. As demonstrated in various studies, serum lipid profile is impaired in hemodialysis patients, especially in those with pre-existing diabetes. This issue plays an apparent role in development and progression of atherosclerosis (5).

Although currently several treatments are available for treatment of lipid disorders, due to the above-mentioned topics including specific conditions in these patients due to chronic disease and mental health problems and consumption of many drugs, finding a way to prevent atherosclerosis progression or serum lipid abnormalities in such patients can have significant role in them.

As noted at the start of this study, consuming food sources whose effects on serum lipids have been confirmed in different studies is one of these ways. Seafood including fish and plankton which contain significant amounts of omega-3 can be a solution for long-term use in such patients and the objective of this study was in fact to confirm this. Geographical situation in the province limit easy access to seafood and ocean resources for all citizens, especially hemodialysis patients. On the other hand, necessary monetary resources may not be available for all patients. Therefore, it is logical that patients consume seafood (if afforded) or take supplements containing omega-3.

But to compare this study with other studies, it is necessary to present the results of some similar studies. Friedman et al, (13) in a study performed in 2006 in the review of 30 studies on the effects of omega-3 in patients found that omega-3 plays a significant role in immune and inflammatory responses, atherosclerosis progression, and blood pressure control.

Rasic et al, in a study on 35 patients who were undergoing hemodialysis for 12 months confirmed the effects of omega-3 on insulin resistance and lowering TNF level (17). Poulia et al on 25 hemodialysis patients confirmed the none effect of omega-3 on inflammatory markers and serum lipids (15).

## Conclusion

The obtained findings suggest that although omega-3 consumption, given the numbers before and after the survey, had some effect, with respect to the fact that ultimate goal is the results of statistical analyses, except for serum total cholesterol (*P *= 0.045), other items did not differ statistically in this study. The only finding was significant decrease in serum total cholesterol.

## Limitations of the study

In this project, there were some limitations including:

1. Limitation in the number of patients interested in participating in the study and taking omega-3.
2. Migration of the patients
3. The probability of mortality at older ages among patients undergoing chronic hemodialysis or lack of regular drug use in older age. Therefore, to avoid such limitations, larger sample should be considered before starting the study.

## Suggestions

The research team, in order to achieve the best results regarding the effects of omega-3 functions on the items discussed in the text, offers the following suggestions:

1. To perform studies with larger sample size
2. Long-term follow-up of patients in order to review and confirm or reject the effects of omega-3 on the studied items.

## Acknowledgments

We thank Mr. Masoud Ahmadi-Masoul and Ms. Mehrangiz Soleimani, the staff nurse of the hemodialysis center and also Imam Reza hospital for their kind contribution to this study.

## Authors’ contribution

All authors contributed equally to design of the research and all authors have read, revised, and approved the final manuscript.

## Ethical considerations

Ethical issues (including plagiarism, misconduct, data fabrication, falsification, double publication or submission, redundancy) have been completely observed by the authors.

## Conflicts of interest

The authors declared no competing interests.

## Funding/Support

This manuscript extracted from MSc thesis 92274 that supported financially by deputy of research of Kermanshah University of Medical Science. The authors are grateful to all who helped in conducting the present Research.
